# 

**Published:** 1908-07

**Authors:** 


Texas Health Officers Association. Official Report.

VOL. XXIV. JULY, 1908. No. 1.

LIBRARY
SURGEON GENERAL'S OFFICE
JUL 20 1908

### TEXAS MEDICAL JOURNAL

"RED BACK"

PUBLISHED AT AUSTIN, TEXAS, BY

F. E. DANIEL, M. D., - - Proprietor

PRINTED BY THE VON BOECKMANN-JONES CO.

Subscription, $1.00 a Year, in Advance. - - - Single Copies, 10 Cents

Entered at the Postoffice at Austin, Texas, as Second-class Mail Matter

